# Bis-Indole Derivatives for Polysaccharide Compositional Analysis and Chiral Resolution of D-, L-Monosaccharides by Ligand Exchange Capillary Electrophoresis Using Borate-Cyclodextrin as a Chiral Selector 

**DOI:** 10.3390/molecules16021682

**Published:** 2011-02-17

**Authors:** Chien-Yuan Kuo, Kuo-Shiang Liao, Yin-Chen Liu, Wen-Bin Yang

**Affiliations:** Genomics Research Center, Academia Sinica, No. 128, Academia Road Section 2, Nankang, Taipei 11529, Taiwan

**Keywords:** monosaccharides, indole, enantioseparation, cyclodextrin, ligand-exchange capillary electrophoresis (LECE)

## Abstract

A series of aldo-bis-indole derivatives (aldo-BINs) was prepared by aromatic *C*-alkylation reactions of aldoses and indole in acetic acid solution. Common monosaccharides such as glucose, mannose, galactose, fucose, xylose, rhamnose, ribose, arabinose and *N*-acetylglucosamine were smoothly derivatized to form the UV absorbing aldo-BINs. The use of a capillary electrophoretic method to separate these novel aldo-BIN derivatives was established. The capillary electrophoresis conditions were set by using borate buffer (100 mM) at high pH (pH 9.0). The limit of determination was assessed to be 25 nM. The enantioseparation of D, L-pairs of aldo-BINs based on chiral ligand-exchange capillary electrophoresis technology was also achieved by using modified hydroxypropyl-β-cyclodextrin as the chiral selector in the presence of borate buffer. This aldose labeling method was applied successfully to the compositional and configurational analysis of saccharides, exemplified by a rapid and efficient method to simultaneously analyze the composition and configuration of saccharides from the medicinal herbs *Cordyceps sinensis* and *Dendrobium huoshanense*.

## 1. Introduction

**Scheme 1:**
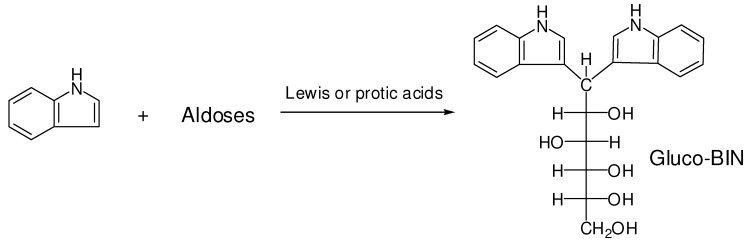
Synthesis of bis(3’-indolyl)alkanes by the *C*-glycosylation of indole with unprotected aldoses [8].

Carbohydrates are one of the most important components of organisms and they are essential materials in many biological processes [1]. Many conjugation methods have been developed by tagging carbohydrates to facilitate their compositional analysis and chiral resolution [2,3]. However, some of these methods are time-consuming and result in low yield due to the many preparation steps required. A series of synthetic methods for the preparation of the bis(3’-indolyl)alkanes using Lewis or protic acids as catalyst have been developed [4,5,6]. In general, the reactions took place by the condensation of two molecules of indole with an aldehyde or ketone [5]. Recently, Sato reported a Lewis acid ([Sc(OTf)_3_], catalyzed synthesis of bis(3’-indolyl)alkanes in aqueous media by the *C*-glycosylation of indole with unprotected aldoses (Scheme 1) [7,8]. Here we used a mild and environmental friendly protic acid (HOAc) for the reaction of unprotected and unmodified aldoses with indole to generate aldo-bis(3’-indolyl)alkanes (aldo-BINs) in a rapid and convenient process. These products should be promising in further application to investigate the composition and stereoconfiguration of saccharides in biological systems. Here, we are interested in the use of aldo-bis(3’-indolyl)alkanes as aldose-tags for the compositional analysis of saccharides by capillary electrophoresis (CE), which has been proven a powerful analytical tool in saccharide analysis [9,10]. In continuation of our studies on a rapid transformation of aldose to its imidazole derivative (aldo-NAIM) using 2,3-naphthalene diamine and catalytic iodine in acetic acid solution [11], we developed a new protocol for the synthesis of various aldo-BINs by direct reductive condensation of aldoses with two moles of indoles in H_2_O/HOAc solution. We also demonstrated that these UV active aldo-BINs are useful for sugar composition analysis.

Chiral resolution is an important topic in analytical chemistry [12,13], because chiral enantiomers have the same physical properties and are thus difficult to separate each other. In addition, optical D-, L-monosaccharide isomers present in Nature lack electric charges and chromophores for chromatographic analysis, so that they are hard to analyze without chemical derivation. CE has been proven as a good tool to resolve D-, L-monosaccharides in recent years [14,15,16] by pre- and in-column introduction of cationic or anionic UV absorbing tags that have been used to analyze enantiomers. The use of borate buffers with chiral selectors has been reported for the enantioseparation of saccharides [17] by derivation with aromatic reagents. These derivatives were separated using the cavity of cyclodextrins (CDs) to form mixed borate complexes in CE chromatography. Stefansson and Novotny firstly reported that enantioseparation of several monosaccharides by reductive amination with 5-aminonaphthalene-2-sulfonic acid, or 4-amino-5-hydroxynaphthalene-2,7-disulfonic acid, and these derivatized D-, L-polyols were enantioseparated by CE as complexes with borate using linear or cyclic dextrins as chiral selectors [18]. Kodama *et al.* used 1-phenyl-3-methyl-5-pyrazolone (PMP) derivatives for chiral resolution of monosaccharides by ligand-exchange capillary electrophoresis (LECE) using borate anion as the central ion presence CDs as the chiral selectors. The electrophoretic patterns of several aldo-PMPs were enantioseparated [19]. 

## 2. Results and Discussion

**Figure 1:**
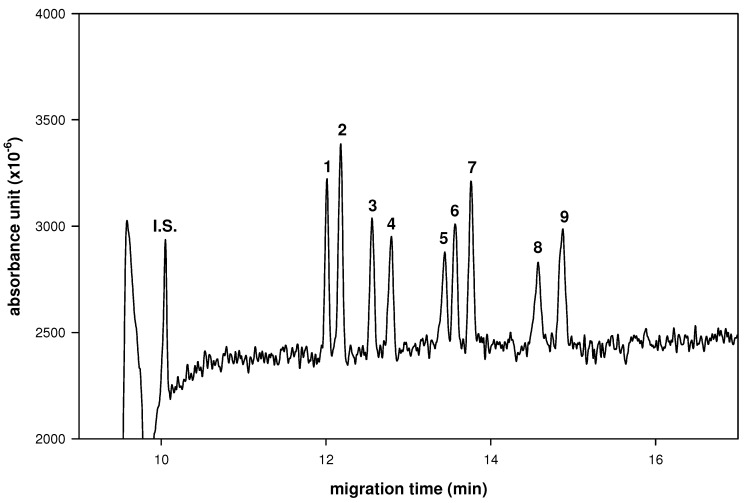
Electrophorogram of aldo-BINs in CE.Peaks: 1 = Rib-BIN; 2 = Rha-BIN; 3 = GlclNAc-BIN; 4 = Xyl-BIN; 5 = Glc-BIN; 6 = Man-BIN; 7 = Ara-BIN; 8 = Gal-BIN; 9 = Fuc-BIN. CE conditions: buffer, 100 mM borate (pH 9.0) contains hydroxypropyl-β-CD (10 mg/mL); applied voltage, 20 kV; uncoated fused-silica capillary, 50/60.2 cm × 50 μm I.D.; sample injection, 3s by pressure (0.5 psi); wavelength, 254 nm; system temperature, 15 °C.

In a preliminary study, we used molecular iodine as catalyst to condense aldoses with aromatic *ortho*-diamines. These aldo-naphthylimidazole (aldo-NAIM) derivatives are separated well in CE for sugar composition analysis [11]. Here, in continuation of our studies, we have synthesized a series of bis(3’-indolyl)alkanes derivatives by direct *C*-glycosylation of indole with aldoses and used them for sugar compositional analysis. Several neutral and amino-containing monosaccharides (rhamnose, xylose, ribose, glucose, mannose, arabinose, fucose, galactose and *N*-acetylglucosamine) were easily derivatized by such chemical labeling. This reaction can be carried out with/without molecular iodine as a catalyst, even though Ji *et al.* had reported that a catalytic amount of molecular iodine improved synthesis of bis(indolyl)methanes at room temperature [6]. Here, we report a simultaneous separation of nine aldo-BINs for saccharide analysis by CE using borate buffers (Figure 1). Not only neutral monosaccharides but also C_6_ deoxyl and amino-containing monosaccharides are separated. In addition, these monosaccharide-BINs are stable at the high pH values (borate buffer, pH 8~10) used for sugar composition analysis by CE.

The resolution and migration time of all derivatives increased, as expected, with increasing borate concentration. The reason might depend on the destabilization of aldo-BIN-borate complex. With the high-pH BGE system, the extents of proton ionization of aldo-BINs and charge/mass ratio would result in differences in migration rates. Considering the resolution and speed, the optimized CE condition was set as 100 mM borate buffer and pH 9.0. These aldo-BINs can be applied to analyze the composition of saccharides in medicinal herbs. Optimization of the separation of nine aldo-BINs was achieved by optimizing the CE conditions, including pH value, temperature, applied voltage and concentration of borate buffer [10]. The migration velocities of derivatives were affected primarily by the extent of five-member diol-borate complexation [19]. The speculative elution order of aldopentose-derivatives was attributed by the orientation of hydroxyl groups at the C3/C4 position; e.g., Ara possessing *cis*-oriented hydroxyl groups was more retarded than Xyl with *trans*-oriented hydroxyl groups. The same behavior could be observed with aldohexoses and deoxyhexoses. As such, the observed elution order of Glc (*trans*) > Man (*trans*) > Gal (*cis*); and Rha (*trans*) > Fuc (*cis*) was similar to that of a previous report [9].

**Figure 2:**
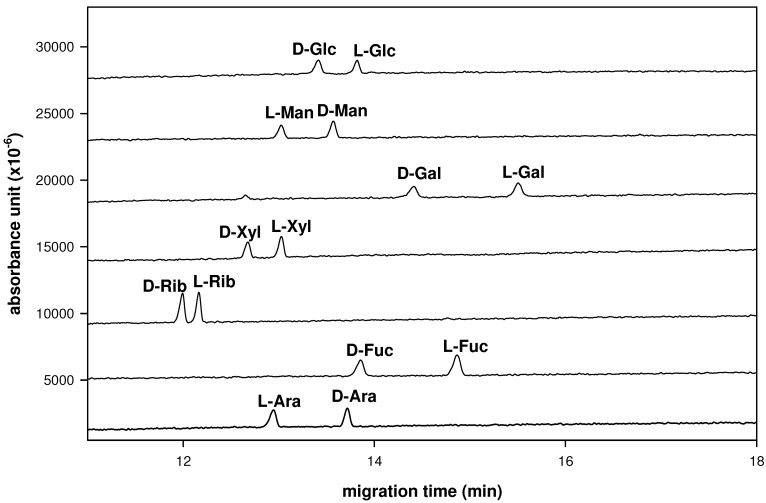
Enantioseparation of seven enantiomeric pairs of indole derivated monosaccharides (aldo-BINs). Conditions: borate buffer (100 mM, pH 9.0) contains hydroxypropyl-β-CD (10 mg/mL); applied voltage, 20 kV (detector at cathode side); uncoated fused-silica capillary, 50 cm (effective length) × 50 μm I.D.; wavelength, 254 nm; system temperature, 15 °C.

Pre-column introduction of cationic or anionic UV absorbing tags have been used to analyze enantiomeric compounds [12,15,20]. We used D-, L-aldoses and a UV absorbing indole to form a series of D-, L-aldo-BIN derivatives by Sato’s method [7,8]. Then, we further investigated that if these UV absorbing D-, L-aldo-BINs can be explored for chiral resolution using the LECE method. The use of borate buffers with chiral selectors has been reported [17,18,19]. The enantioseparation conditions were similar to Stefansson’s method whereby these derivatized D-, L-polyols were enantioseparated by CE as complexes with borate and linear or cyclic dextrins used as a chiral selector [18]. We determined these D-, L-polyol-BINs by using hydroxypropyl-β-CD as selector ligand in LECE to investigate the ability of enantioseparation for these new derivatives. We found that using a borate buffer system as mobile phase, a series of D-, L-aldo-BINs can be separated well in CE for monosaccharide enantioseparation analysis (Figure 2). The ability to enantioseparate seven pairs of D-, L-aldo-BIN derivatives was tested on the each enantiomeric pair, individually. The electrophoretic experiments were set in 10 mg/mL of hydroxypropyl-β-CD, 100 mM of borate buffer at pH 9.0 with an uncoated fused-silica capillary. Each D-, L-monosaccharide pair could be identified, even if a short capillary column was used. 

Enantioseparation depends on the stability of the ternary space complex when chiral ligands are used in CE [12]. Therefore, a high concentration of borate anion in the CE system contributed to the stability of the borate complexes. It seems that the electrophoretic pattern of aldo–BINs depends on the ionic strength of the borate solution, which affects the running current during the electrophoresis. The longer migration time at the higher ionic concentration may be attributed to an increase in the viscosity and migration time. However, to increase the peak’s resolution by the enhancement of the interaction between the enantiomers (D-, L-aldo-BIN) and the hydroxypropyl-β-CD complex, while maintaining a moderately short migration time, the appropriate CE conditions were set at hydroxypropyl-β-CD (10 mg/mL) with 100 mM borate buffer and the migration time was shortened to under 20 min. Consequently, the optimum conditions for both high resolution and moderately short migration time consisted of 10 mg/mL of hydroxypropyl-β-CD with 100 mM borate buffer at pH 9.0, 15 °C, 20 kV and 254 nm. To elucidate the mechanism of enantioseparation, the migration order of D-aldo-BINs and L-aldo-BINs were confirmed for the aldo-BINs used in this study (Figure 1). Typically the D-forms migrated faster than the L-forms (except for mannose and arabinose, Figure 2) when hydroxypropyl-β-CD was used as chiral selector. The most likely explanation for this migration behavior is that the separation of the enantiomers is due to formation of diastereomeric ternary complexes, however, the details of the mechanism of interaction between aldo-BINs and the chiral selector are unclear. 

**Figure 3:**
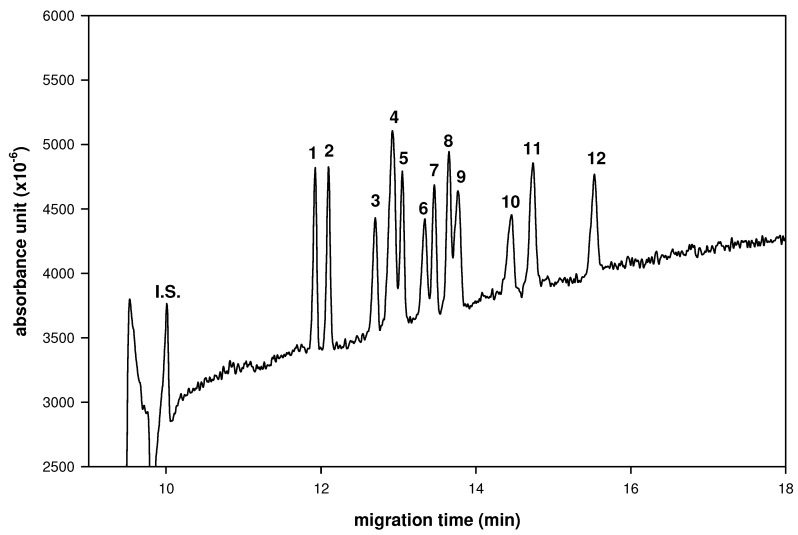
Simultaneous enantioseparation of seven enantiomeric pairs of indole derivatized monosaccharides. Peaks: 1 = D-Rib-BIN; 2 = L-Rib-BIN; 3 = D-Xyl-Bin; 4 = L-Man-BIN + L-Ara-BIN; 5 = L-Xyl-BIN; 6 = D-Glc-BIN; 7 = D-Man-BIN; 8 = D-Ara-BIN; 9 = L-Glc-BIN + D-Fuc-BIN; 10 = D-Gal-BIN; 11 = L-Fuc-BIN; 12 = L-Gal-BIN. CE conditions: borate buffer (100 mM, pH 9.0) contains hydroxypropyl-β-CD (10 mg/mL); applied voltage, 20 kV (detector at cathode side); uncoated fused-silica capillary, 50 cm (effective length) × 50 μm I.D.; wavelength, 254 nm; system temperature, 15 °C.

Simultaneous analysis of seven common saccharide pairs of D-, L-aldo-BINs was investigated by LECE using borate as a central ion with hydroxypropyl-β-CD as a chiral selector ligand (Figure 3). The enantiomeric pairs of D-, L-aldo-BINs were separated from each other, however, several peaks corresponding to different types of aldo-BINs overlap slightly and they can be separated by further optimization of the CE analysis conditions. This result indicates that LECE is applicable to separate enantiomeric pairs of monosaccharides in the same time, which is attributed to the formation of a mixed of diastereomeric aldo–BIN–hydroxypropyl-β-CD ternary complex with the various types of aldo-BINs. 

**Figure 4:**
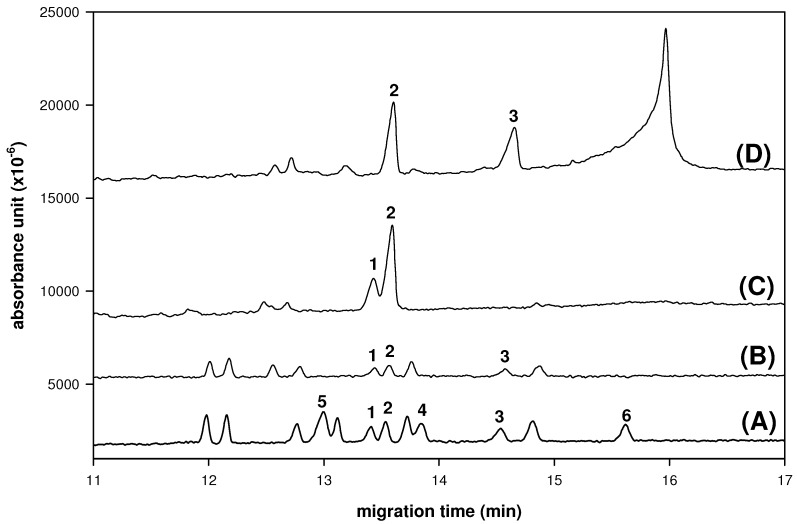
Electrophorograms of composition and configuration analysis of polysaccharide hydrolysate from medicinal herbs. (A) Enantioseparation of D, L pairs of seven kinds of aldo-BINs. (B) Standards of nine D-aldo-BINs for monosaccharide compositional analysis. (C) The polysaccharide hydrolysate from *Dendrobium huoshanen.* (D) The polysaccharide hydrolysate from *Cordyceps sinensis*. Peaks: 1 = D-Glc-BIN; 2 = D-Man-BIN; 3 = D-Gal-BIN; 4 = L-Glc-BIN; 5 = L-Man-BIN; 6 = L-Gal-BIN. CE conditions: buffer, 100 mM borate (pH 9.0) contains hydroxypropyl-β-CD (10 mg/mL); applied voltage, 20 kV (detector at cathode side); uncoated fused-silica capillary, 50 cm (effective length) × 50 μm I.D.; sample injection, 3s by pressure (0.5 psi); wavelength, 254 nm; system temperature, 15 °C.

Because of the potential of aldo-BINs in saccharides compositional and chiral resolution in medicinal herbs by CE analysis we propose that the aldo-BINs derivative might be applicable to the simultaneous analysis of the composition and chiral resolution of the saccarides. Indeed, the polysaccharides from *Cordyceps sinensis* (*C. sinensis*) and *Dendrobium huoshanens* (*D. huoshanens*), which is a tonic medicinal herb with immunomodulatory functions to stimulate some growth factors *in vitro* [21,22,23,24,25] have been determined in this study (Figure 4). The hydrolysate of polysaccharides in *Dendrobium huoshanen* was composed with D-mannose (75.2%) and D-glucose (24.8%) and the hydrolysate of polysaccharides in *Cordyceps sinensis* was composed of D-mannose (53.0%) and D-galactose (47.0%), respectively.

## 3. Experimental

### 3.1. General

D, L-Glucose (Glc), D, L-galactose (Gal), D, L-mannose (Man), D, L-arabinose (Ara), D, L-ribose (Rib), D, L-xylose (Xyl), D, L-fucose (Fuc), L-rhamnose (Rha), *N*-acetyl D-glucosamine (GlcNAc), hydroxypropyl-β-CD and 2-naphthol (used as the internal standard, IS) were purchased from Sigma-Aldrich (St. Louis, MO, USA). Indole, acetic acid and iodine were purchased from Acros (Morris Plains, NJ, USA). Disodium tetraborate (Na_2_B_4_O_7_), hydrochloric acid (HCl) and sodium hydroxide (NaOH) were obtained from Merck (Darmstadt, Germany) and all materials are analytical grade. Milli-Q water (Millipore, Bedford, MA, USA) was used for the preparation of buffer and related aqueous solution. Analytical instruments: The NMR spectra were recorded on Bruker 600 MHz NMR spectrometer (Bruker BioSpin GmbH, Rheinstetten, Germany) with 5 mm Cryoprobe DCI ^1^H/^13^C. The MALDI-TOF mass spectrometer which was used to acquire the spectra was a Voyager Elite (Applied Biosystems, Foster City, CA. USA), equipped with a nitrogen pulsed laser (337 nm). The accelerating voltage was set at 20 kV in a positive ion mode. Spectra from 80–100 laser shots were accumulated to obtain the final spectrum. The CE system is described as section 3.3 in below.

### 3.2. Preparation of aldo-BINs

**Figure 5:**
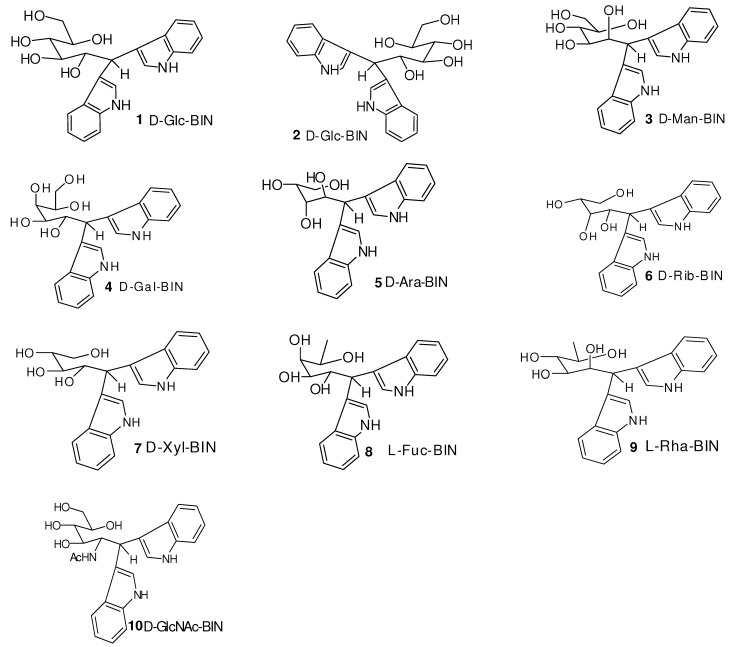
The chemical structures of D-, L-aldo-BIN.

Typically, a solution of aldose (54.0 mg, 0.3 mmol) and indole (87.9 mg, 0.8 mmol) was stirred with iodine (0.5 mg, 10 mol%) in AcOH (1.0 mL) for 12~16 h at 50 °C. The reaction was completed as indicated by the TLC analysis. The reaction mixture was concentrated under reduced pressure. The residue was purified by flash column chromatography on silica gel (9:1 CHCl_3_/MeOH) to give the aldo-BINs in 80~95% yields (Figure 5). 

*(2R,3R,4R,5S)-6,6-di(1H-Indol-3-yl)hexane-1,2,3,4,5-pentaol*** [8] **C_22_H_24_N_2_O_5_ **1**: brown solid; [α]^20^_D_ +51 (c 1.0, MeOH); *Rf* 0.25 (8:2 CHCl_3_/MeOH); ^1^H-NMR (MeOD-*d_4_*, 600 MHz) δ 7.74 (1 H, d, *J* = 8.0 Hz, ArH), 7.61 (1 H, d, *J* = 8.0 Hz, ArH), 7.38 (1 H, s, ArH), 7.30 (1 H, d, *J* = 12.7 Hz, ArH), 7.28 (1 H, d, *J* = 12.7 Hz, ArH) 7.09 (1 H, s, ArH), 7.04 (1 H, d, *J* = 14.7 Hz, ArH), 7.03 (1 H, d, *J* = 14.8 Hz, ArH), 7.00 (1 H, d, *J* = 8.0 Hz, ArH), 6.92 (1 H, d, *J* = 8.0 Hz, Ar H), 4.96 (1 H, d, *J* = 7.8 Hz, H-6), 4.69 (1 H, dd, *J* = 7.8, 3.6 Hz, H-5), 3.92 (1 H, dd, *J* = 3.6, 2.3 Hz, H-4), 3.74 (1 H, dd, *J* = 7.3, 2.3 Hz, H-3), 3.70–3.67 (2 H, m, H-2, H-1a), 3.54 (1 H, dd, *J* = 12.3, 6.9 Hz, H-1b); ^13^C-NMR (MeOD-*d_4_*, 150 MHz) δ 138.1, 138.0, 129.3, 128.3, 124.3, 124.2, 122.3, 122.2, 120.31, 120.29, 119.56, 119.50, 118.0, 116.6, 112.24, 112.19, 77.6, 75.3, 73.3, 71.2, 64.7, 38.0; MS (MALDI) found: m/z = 419.105 [M + Na]^+^.

*(2S,3S,4S,5R)-6,6-di(1H-Indol-3-yl)hexane-1,2,3,4,5-pentaol* C_22_H_24_N_2_O_5_ **2**: brown solid; [α]^20^_D_ –50 (c 1.0, MeOH); *Rf* 0.25 (8:2 CHCl_3_/MeOH); ^1^H-NMR (MeOD-*d_4_*, 600 MHz) δ 7.71 (1 H, d, *J* = 7.9 Hz, ArH), 7.60 (1 H, d, *J* = 7.9 Hz, ArH), 7.40 (1 H, s, ArH), 7.30 (1 H, d, *J* = 11.6 Hz, ArH), 7.29 (1 H, d, *J* = 11.6 Hz, ArH), 7.12 (1 H, s, ArH), 7.03 (1 H, t, *J* = 6.9 Hz, ArH), 7.02 (1 H, t, *J* = 6.9 Hz, ArH), 6.95 (1 H, t, *J* = 7.6 Hz, ArH), 6.91 (1 H, t, *J* = 7.6 Hz, ArH), 4.93 (1 H, d, *J* = 7.9 Hz, H-6), 4.67 (1 H, dd, *J* = 7.9, 3.4 Hz, H-5), 3.92 (1 H, dd, *J* = 3.4, 2.0 Hz, H-4), 3.72 (1 H, dd, *J* = 7.2, 2.0 Hz. H-3), 3.67–3.64 (2 H, m, H-2, H-1a), 3.50 (1 H, dd, *J* = 12.3, 6.9 Hz, H-1b); ^13^C-NMR (MeOD-*d_4_*, 150 MHz) δ 138.3, 138.2, 129.3, 128.5, 124.3, 124.2, 122.3, 122.2, 120.4, 120.3, 119.6, 119.5, 118.0, 116.7, 112.3, 112.2, 77.7, 75.4, 73.3, 71.2, 64.7, 38.2; MS (MALDI) found: m/z = 419.115 [M + Na]^+^.

*(2R,3R,4R,5R)-6,6-di(1H-Indol-3-yl)hexane-1,2,3,4,5-pentaol* C_22_H_24_N_2_O_5_ **3**: brown solid; [α]^20^_D_ –18 (c 1.1, MeOH); *Rf* 0.28 (8:2 CHCl_3_/MeOH); ^1^H-NMR (MeOD-*d_4_*, 600 MHz) δ 7.70 (1 H, d, *J* = 8.0 Hz, ArH), 7.56 (1 H, d, *J* = 8.0 Hz, ArH), 7.34 (1 H, s, ArH), 7.30 (2 H, t, *J* = 7.9 Hz, ArH), 7.19 (1 H, s, ArH), 7.04 (2 H, t, *J* = 7.2 Hz, ArH), 6.94 (1 H, t, *J* = 7.3 Hz, ArH), 6.91 (1 H, t, *J* = 7.3 Hz, ArH), 5.15 (1 H, d, *J* = 3.0 Hz, H-6), 4.52 (1 H, dd, *J* = 8.5, 3.0 Hz, H-5), 3.92 (1 H, d, *J* = 7.9 Hz, H-3), 3.78 (1 H, d, *J* = 8.5 Hz, H-4), 3.73 (1 H, dd, *J* = 11.2, 3.6 Hz, H-1a), 3.65 (1 H, m, H-2), 3.56 (1 H, dd, *J* = 11.2, 3.6 Hz, H-1b); ^13^C-NMR (MeOD-*d_4_*, 150 MHz) δ 138.3, 137.9, 129.9, 128.5, 125.3, 124.4, 122.2, 122.1, 120.5, 120.2, 119.5, 119.4, 119.1, 115.2, 112.2, 112.1, 75.4, 73.4, 71.7, 71.4, 65.1, 36.6; MS (MALDI) found: m/z = 419.116 [M + Na]^+^.

*(2R,3S,4R,5S)-6,6-di(1H-Indol-3-yl)hexane-1,2,3,4,5-pentaol* C_22_H_24_N_2_O_5_** 4**: brown solid; [α]^20^_D_ +30 (c 1.1, MeOH); *Rf* 0.24 (8:2 CHCl_3_/MeOH); ^1^H-NMR (MeOD-*d_4_*, 600 MHz) δ 7.67 (1 H, d, *J* = 7.9 Hz, ArH), 7.64 (1 H, d, *J* = 7.9 Hz, ArH), 7.33 (1 H, s, ArH), 7.29 (1 H, d, *J* = 13.1 Hz, ArH), 7.27 (1 H, d, *J* = 13.1 Hz, ArH), 7.23 (1 H, s), 7.01 (2 H, t, *J* = 7.9 Hz, ArH), 6.92 (1 H, t, *J* = 7.3 Hz, ArH), 6.91 (1 H, t, *J* = 7.3 Hz, ArH), 4.90 (1 H, d, *J* = 10.3 Hz, H-6), 4.85 (1 H, dd, *J* = 10.3, 6.5 Hz, H-5), 3.89 (1 H, td, *J* = 6.5, 0.8 Hz, H-4), 3.76–3.72 (2 H, m, H-3, H-1a), 3.62–3.57 (2 H, m, H-2, H-1b); ^13^C-NMR (MeOD-*d_4_*, 150 MHz) δ 138.3, 138.1, 129.2, 128.6, 123.9, 123.8, 122.21, 122.18, 120.4 (2 ×), 119.5, 119.4, 118.2, 117.7, 112.2 (2 ×), 73.7, 72.1, 71.9, 71.8, 65.1, 38.4; MS (MALDI) found: m.z = 419.108 [M + Na]^+^.

*(2R,3R,4R)-5,5-di(1H-Indol-3-yl)pentane-1,2,3,4-tetraol* C_21_H_22_N_2_O_4_** 5**: brown solid; [α]^20^_D_ –51 (c 1.0, MeOH); *Rf* 0.39 (8:2 CHCl_3_/MeOH); ^1^H-NMR (MeOD-*d_4_*, 600 MHz) δ 7.67 (1 H, d, *J* = 8.0 Hz, ArH), 7.63 (1 H, d, *J* = 8.0 Hz, ArH), 7.32 (1 H, s, ArH), 7.28 (1 H, d, *J* = 10.6 Hz, ArH), 7.27 (1 H, d, *J* = 10.6 Hz, ArH), 7.19 (1 H, s, ArH), 7.02 (2 H, t, *J* = 7.4 Hz, ArH), 6.93 (1 H, t, *J* = 7.5 Hz, ArH), 6.91 (1 H, t, *J* = 7.5 Hz, ArH), 4.90 (1 H, br, H-4), 4.8 (1 H, d, *J* = 10.1 Hz, H-5), 3.77–3.75 (2 H, m, H-3, H-1a), 3.60 (1 H, dd, *J* = 7.1, 4.6 Hz, H-2), 3.54 (1 H, dd, *J* = 12.1, 7.1 Hz, H-1b); ^13^C-NMR (MeOD-*d_4_*, 150 MHz) δ 138.3, 138.1, 129.3, 128.6, 123.9, 123.8, 122.24, 122.19, 120.4 (2 ×), 119.54, 119.46, 118.1, 117.8, 112.2 (2 ×), 73.8, 73.7, 72.8, 65.2, 38.3; MS (MALDI) found: m/z = 389.077 [M + Na]^+^.

*(2R,3R,4S)-5,5-di(1H-Indol-3-yl)pentane-1,2,3,4-tetraol* C_21_H_22_N_2_O_4_** 6**: brown solid; [α]^20^_D_ +58 (c 1.0, MeOH); *Rf* 0.46 (8:2 CHCl_3_/MeOH); ^1^H-NMR (MeOD-*d_4_*, 600 MHz) δ 7.66 (1 H, d, *J* = 8.0 Hz, ArH), 7.61 (1 H, d, *J* = 8.0 Hz, ArH), 7.37 (1 H, s, ArH), 7.31 (1 H, d, *J* = 11.4 Hz, ArH), 7.30 (1 H, d, *J* = 11.4 Hz, ArH), 7.12 (1H, s, ArH), 7.05 (1 H, t, *J* = 7.4 Hz, ArH), 7.04 (1 H, t, *J* = 7.4 Hz, ArH), 6.94 (1 H, t, *J* = 7.3 Hz, ArH), 6.93 (1 H, t, *J* = 7.3 Hz, ArH), 5.16 (1 H, d, *J* = 3.1 Hz, H-5), 4.42 (1 H, dd, *J* = 8.5, 3.1 Hz, H-4), 3.88 (1 H, dd, *J* = 9.5, 5.8 Hz, H-2), 3.80 (1 H, dd, *J* = 11.4, 3.7 Hz, H-1a), 3.69–3.64 (2 H, m, H-3, H-1b); ^13^C-NMR (MeOD-*d_4_*, 150 MHz) δ 138.3, 137.8, 129.9, 128.4, 125.3, 124.6, 122.2, 122.1, 120.4, 120.1, 119.5, 119.5, 118.7, 115.1, 112.3, 112.1, 77.7, 75.3, 73.9, 64.1, 36.8; MS (MALDI) found: m/z = 389.076 [M + Na]^+^. 

*(2R,3S,4S)-5,5-di(1H-Indol-3-yl)pentane-1,2,3,4-tetraol* C_21_H_22_N_2_O_4_** 7**: brown solid; [α]^20^_D_ +60 (c 1.1, MeOH); *Rf* 0.43 (8:2 CHCl_3_/MeOH); ^1^H-NMR (MeOD-*d_4_*, 600 MHz) δ 7.71 (1 H, d, *J* = 8.0 Hz, ArH), 7.59 (1 H, d, *J* = 8.0 Hz, ArH), 7.37 (1 H, s, ArH), 7.28 (1 H, d, *J* = 8.4 Hz, ArH), 7.27 (1 H, d, *J* = 8.4 Hz, ArH), 7.10 (1 H, s, ArH), 7.03 (1 H, t, *J* = 7.6 Hz, ArH), 7.02 (1 H, t, *J* = 7.6 Hz, ArH), 6.95 (1 H, t, *J* = 7.7 Hz, ArH), 6.90 (1 H, t, *J* = 7.7 Hz, ArH), 4.93 (1 H, d, *J* = 8.0 Hz, H-5), 4.63 (1 H, dd, *J* = 8.0, 3.3 Hz, H-4), 3.81 (1 H, dd, *J* = 10.2, 4.5 Hz, H-1a), 3.67 (1 H, t, *J* = 3.6 Hz, H-2), 3.62–3.53 (2 H, m, H-3, H-1b); ^13^C NMR (MeOD-*d_4_*, 150 MHz) δ 138.2, 138.1, 129.3, 128.5, 124.3, 124.1, 122.3, 122.2, 120.34, 120.29, 119.6, 119.5, 118.1, 117.0, 112.3, 112.2, 76.3, 75.2, 72.6, 64.7, 38.0; MS (MALDI) found: m/z = 389.071 [M + Na]^+^.

*(2S,3R,4S,5R)-1,1-di(1H-Indol-3-yl)hexane-2,3,4,5-tetraol* C_22_H_24_N_2_O_4_** 8**: brown solid; [α]^20^_D_ –34 (c 1.0, MeOH); *Rf* 0.55 (8:2 CHCl_3_/MeOH); ^1^H-NMR (MeOD-*d_4_*, 600 MHz) δ 7.69 (1 H, d, *J* = 8.0 Hz, ArH), 7.65 (1 H, d, *J* = 8.0 Hz, ArH), 7.30 (1 H, s, ArH), 7.28 (1 H, d, *J* = 14.4 Hz, ArH), 7.26 (1 H, d, *J* = 14.4 Hz, ArH), 7.21 (1 H, s, ArH), 7.03 (2 H, t, *J* = 7.0 Hz, ArH), 6.93 (1 H, t, *J* = 7.3 Hz, ArH), 6.91 (1 H, t, *J* = 7.3 Hz, ArH), 4.90 (1 H, br, H-2), 4.85 (1 H, d, *J* = 10.3 Hz, H-1), 4.01 (1 H, dd, *J* = 10.3, 2.3 Hz, H-3), 3.71 (1 H, d, *J* = 6.5 Hz, H-5), 3.49 (1 H, dd, *J* = 7.5, 2.3 Hz, H-4), 1.12 (3 H, d, *J* = 6.5 Hz, CH_3_); ^13^C-NMR (MeOD-*d_4_*, 150 MHz) δ 138.2, 138.1, 129.3, 128.6, 123.9, 123.8, 122.2, 122.2, 120.5 (2 ×), 119.5, 119.5, 118.2, 117.8, 112.2 (2 ×), 75.9, 74.0, 71.9, 67.8, 38.4, 20.1; MS (MALDI) found: m/z = 403.114 [M + Na]^+^.

*(2R,3R,4R,5R)-1,1-di(1H-Indol-3-yl)hexane-2,3,4,5-tetraol* C_22_H_24_N_2_O_4_** 9**: brown solid; [α]^20^_D_ +120 (c 1.0, MeOH); *Rf* 0.53 (8:2 CHCl_3_/MeOH); ^1^H-NMR (MeOD-*d_4_*, 600 MHz) δ 7.71 (1 H, d, *J* = 8.0 Hz, ArH), 7.60 (1 H, d, *J* = 8.0 Hz, ArH), 7.34 (1 H, s, ArH), 7.31 (2 H ,t, *J* = 8.2 Hz, ArH), 7.18 (1 H, s, ArH), 7.06 (2 H, t, *J* = 7.9 Hz, ArH), 6.95 (1 H, t, *J* = 7.7 Hz, ArH), 6.93 (1 H, t, *J* = 7.7 Hz, ArH), 5.17 (1 H, d, *J* = 3.2 Hz, H-1), 4.55 (1 H , dd, *J* = 8.4, 3.2 Hz, H-2), 3.85 (1 H, d, *J* = 8.4 Hz, H-3), 3.78 (1 H, m, H-5), 3.68 (1 H, d, *J* = 7.5 Hz, H-4), 1.18 (3 H, d, *J* = 6.3 Hz, CH_3_); ^13^C NMR (MeOD-*d_4_*, 150 MHz) δ 138.2, 137.8, 129.8, 128.4, 125.3, 124.3, 122.2, 122.1, 120.5, 120.2, 119.5, 119.4, 119.0, 115.2, 112.2, 112.1, 75.6, 75.1, 71.5, 69.3, 36.6, 20.6; MS (MALDI) found: m/z = 419.105 [M + Na]^+^.

*N-((2S,3R,4R,5R)-3,4,5,6-Tetrahydroxy-1,1-di(1H-indol-3-yl)hexane-2-yl)acetamide* C_24_H_27_N_3_O_5_** 10**: brown solid; [α]^20^_D_ –37 (c 1.1, MeOH); *Rf* 0.55 (8:2 CHCl_3_/MeOH); ^1^H-NMR (MeOD-*d_4_*, 600 MHz) δ 7.73 (1 H, d, *J* = 8.0 Hz, ArH), 7.55 (1 H, d, *J* = 8.0 Hz, ArH), 7.27–7.24 (3 H, m, ArH), 7.21 (1 H, s, ArH), 7.02 (1 H, t, *J* = 7.4 Hz, ArH), 7.00 (1 H, t, *J* = 7.4 Hz, ArH), 6.94 (1 H, t, *J* = 7.4 Hz, ArH), 6.89 (1 H, t, *J* = 7.4 Hz, ArH), 5.06 (1 H, dd, *J* = 10.8, 4.8 Hz, H-2), 4.87 (1 H, d, *J* = 10.8 Hz, H-1), 4.07–4.03 (2 H, m, H-3, H-4), 3.87–3.84 (2 H, m, H-5, H-6a), 3.65 (1 H, dd, *J* = 5.9, 2.6 Hz, H-6b); ^13^C-NMR (MeOD-*d_4_*, 150 MHz) δ 173.7, 138.3, 138.1, 129.1, 128.5, 124.3, 122.8, 122.3, 122.1, 120.5, 120.0, 199.6, 119.4, 118.4, 117.8, 122.3, 112.1, 83.2, 74.3, 74.0, 72.7, 55.2, 37.5, 22.7; MS (MALDI) found: m/z = 442.149 [M + Na]^+^.

### 3.3. CE system

A Beckman P/ACE System MDQ (Fullerton, CA, USA) equipped with a filter UV detector and a liquid-cooling device was used. The separation of aldo-BINs was carried out in an uncoated-silica capillary (50 cm length × 50 μm i.d.; Polymicro Technologies, USA). The background electrolyte (BGE) was borate buffer (100 mM, pH 9.0). The samples were injected by pressure (0.5 psi for 3s) at the anodic end of the capillary and a constant voltage of +20 kV was applied during analyses. For enantioseparation of seven pairs of racemic aldo-BINs, the analyses were achieved by using BGE consisted of borate buffer (100 mM, pH 9.0) and hydroxypropyl-β-CD (10 mg/mL) as the chiral selector in a 50 cm capillary (effective length) at 20 kV. After CE analysis the each new sample running was conditioned with 0.1 M NaOH for 5 min, water for 3 min and BGE for another 5 min.

### 3.4. Optimization of the separation of aldo-BIN derivatives

Simultaneous determination of nine aldo-BINs was achieved using optimized CE conditions (Figure 1). Effects of concentration of borate buffer, chiral selector, separation voltage, temperature and pH were studied to decide the optimal separation conditions. The resolution and migration time of all derivatives increased in expectation with increasing of borate concentration. The reason was depend on the destabilization of aldo-BIN-borate complex. With the high-pH BGE system, the extents of proton ionization of aldo-BINs and charge/mass ratio would result in the difference of migration velocities. Considering the resolution and migration time, the optimizing CE condition was set as borate buffer (100 mM), hydroxypropyl-β-CD (10 mg/mL), 20 kV, 15 °C and its pH was 9.0.

## 4. Conclusions

We have achieved a simple and efficient process for saccharide labeling using indole as a tagging agent. Various aldoses react readily with indole to form the corresponding aldo-BINs in high yield. In comparison with the reductive amination of saccharides, this reaction is easier to operate and more environmentally friendly. Here we demonstrate that not only the compositional analysis but also the enantioseparation of D-, L-monosaccharides is facilitated by incorporating a chiral selector in borate buffer. Several kinds of D-, L-aldo-BIN pairs can be enantioseparated distinctively in this LECE system. The present CE method also allows simultaneous chiral resolution and composition analysis of polysaccharides from medicinal herbs. Here, D-, L-aldo-BINs in CE chromatography provides a rapid method for identification of saccharides’ D-, L-configuration, which should be promising in further application to investigate the composition and stereoconfiguration of saccharides in medicinal herbs. This is a first example of the use of aldo–BINs for compositional analysis of polysaccharides and the enantioseparation of monosaccharides in LECE analysis.
